# Broad-scale redistribution of mRNA abundance and transcriptional machinery in response to growth rate in *Salmonella enterica* serovar Typhimurium

**DOI:** 10.1099/mgen.0.000127

**Published:** 2017-08-04

**Authors:** Andrew D. S. Cameron, Shane C. Dillon, Carsten Kröger, Laurens Beran, Charles J. Dorman

**Affiliations:** ^1^​ Institute of Microbial Systems and Society, University of Regina, Regina, SK, S4S 0A2, Canada; ^2^​ Department of Biology, University of Regina, Regina, SK, S4S 0A2, Canada; ^3^​ School of Biological Sciences, Dublin Institute of Technology, Kevin Street, Dublin 8, Ireland; ^4^​ Department of Microbiology, Moyne Institute of Preventive Medicine, Trinity College Dublin, Dublin 2, Ireland

**Keywords:** bacterial chromosome, transcriptomics, chromatin immunoprecipitation, Sigma factors, RNA polymerase

## Abstract

We have investigated the connection between the four-dimensional architecture of the bacterial nucleoid and the organism's global gene expression programme. By localizing the transcription machinery and the transcriptional outputs across the genome of the model bacterium *Salmonella enterica* serovar Typhimurium at different stages of the growth cycle, a surprising disconnection between gene dosage and transcriptional output was revealed. During exponential growth, gene output occurred chiefly in the Ori (origin), Ter (terminus) and NSL (non-structured left) domains, whereas the Left macrodomain remained transcriptionally quiescent at all stages of growth. The apparently high transcriptional output in Ter was correlated with an enhanced stability of the RNA expressed there during exponential growth, suggesting that longer mRNA half-lives compensate for low gene dosage. During exponential growth, RNA polymerase (RNAP) was detected everywhere, whereas in stationary phase cells, RNAP was concentrated in the Ter macrodomain. The alternative sigma factors RpoE, RpoH and RpoN were not required to drive transcription in these growth conditions, consistent with their observed binding to regions away from RNAP and regions of active transcription. Specifically, these alternative sigma factors were found in the Ter macrodomain during exponential growth, whereas they were localized at the Ori macrodomain in stationary phase.

## Abbreviations

ChIP, chromatin immunoprecipitation; ChIP-chip, chromatin immunoprecipitation-microarray; NSL, non-structured left; NSR, non-structured right; Ori, origin; RNAP, RNA polymerase; RNA-seq, RNA sequencing; Ter, terminus.

## Data Summary

1. Microarray datasets have been deposited at the National Center for Biotechnology Information (NCBI) Gene Expression Omnibus (GEO) database; accession numbers GSE97283 (chromatin immunoprecipitation-microarray analysis data; url - https://www.ncbi.nlm.nih.gov/geo/query/acc.cgi?acc=GSE97283) and GSE97161 (transcriptome data; url - https://www.ncbi.nlm.nih.gov/geo/query/acc.cgi?acc=GSE97161).

2. RNA sequencing data analysed in this manuscript was previously published at DOI: http://dx.doi.org/10.1016/j.chom.2013.11.010. Raw reads (.fastq file format) and normalized ‘IGB files’ (.gr file format) are available at NCBI GEO database; accession number GSE49829.

## Impact Statement

This study employs a top-down analysis of genome-wide transcriptional activity to identify broad geographical and temporal patterns of gene expression and transcription machinery distribution around the *Salmonella enterica* chromosome. The multicopy gene effect created by chromosome replication is superseded by regional differences in transcription activity, including the existence of a consistently low activity macrodomain and the apparent evolution of extended RNA half-lives near the replication terminus that may counterbalance multicopy effects nearer the origin of replication. Most surprising is the discovery that inactive sigma factors bind to DNA away from RNA polymerases (RNAPs), then reposition to locations diametrically opposed to RNAPs as transcriptional activity shifts through growth. This research hints at large-scale spatial organization of a bacterial chromosome consistent with the macrodomains described in *Escherichia coli*. Charting the dynamic nature of chromosome spatial structure is an important step in identifying the evolutionary constraints that shape bacterial chromosomes and will contribute to the rational design of synthetic chromosomes.

## Introduction

The bacterial chromosome is more than simply a carrier of genetic information. It is becoming apparent that a blueprint for the gene expression programme of the cell may be written into the geography and architecture of the folded chromosome [1]. This programme possesses both spatial and temporal dimensions, making it likely that the positions of genes within the genome, together with the timing of their expression, are important determinants of the programme's operation [1, 2]. This proposal is supported by the finding that the needs of gene regulation at the level of transcription influence the relative locations of regulatory genes and of the structural genes, operons and regulons that they control [2–10].

**Fig. 1.**
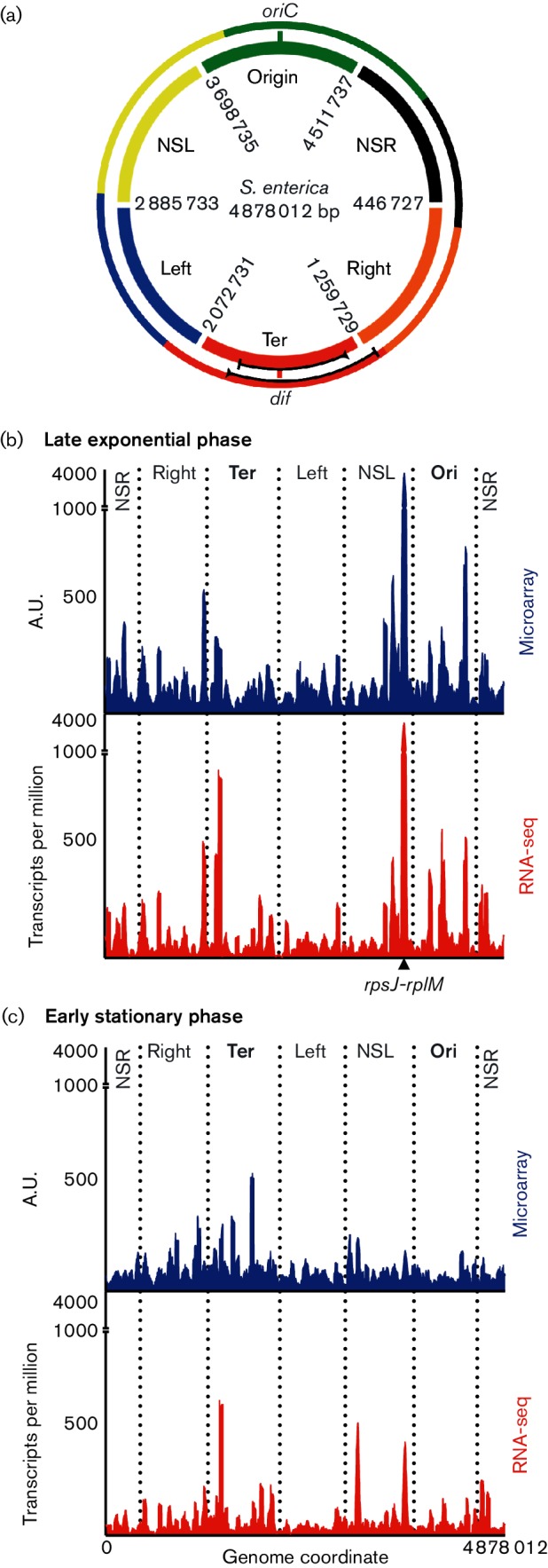
Organization of and transcriptional activity in the *S.* *enterica* serovar Typhimurium SL1344 chromosome. (a) Macrodomain organization of the *E. coli* MG1655 chromosome (outer ring; adapted from Valens *et al*. [24]) compared to the *S. enterica* serovar Typhimurium SL1344 chromosome divided into six equal sized portions (inner ring). Coordinates of the *S*. *enterica* serovar Typhimurium chromosome are indicated. The origin (*oriC*) and terminus (*dif*) site of chromosome replication are indicated; the region and direction of inversion that differs between *E. coli* and *S.* *enterica* serovar Typhimurium is indicted by the black lines in the Ter domains. (b, c) Distribution of transcription across the *S.* *enterica* serovar Typhimurium chromosome in late exponential phase growth (b) and early stationary phase (c), as quantified by whole-genome tiled microarrays (blue) and RNA-seq (red). Microarray data are expressed in arbitrary units (A.U.) by normalizing the sum of expression values to a total of one million, making the genome-wide gene expression profile comparable to the transcripts per million quantification employed in RNA-seq analysis.

The chromosome of the model bacterium *Salmonella enterica* serovar Typhimurium has been studied for many decades as an aid to understanding the link between gene position and bacterial physiology [11–17]. An advantage of studies that focus on *S.* *enterica* serovar Typhimurium is that the chromosome of this pathogen is closely related in size, gene content and gene order to the chromosome of the commensal bacterium *Escherichia coli*, arguably the best understood of all living cells [18]. The discovery that the *E. coli* chromosome is divided into a small number of distinct domains was an important advance in our understanding of bacterial chromosomal architecture [19–24]. An analysis of permissible and non-permissible random intrachromosomal interactions performed using the bacteriophage lambda Int-mediated site-specific recombination apparatus indicates that there are four macrodomains designated Ori (origin), Right, Ter (terminus) and Left, with Ori and Right being separated by the non-structured right (NSR) domain, while the non-structured left (NSL) domain is interposed between Left and Ori [24–26] (Fig. 1a).

The bidirectional replication of the chromosome creates two replichores (Left and Right), and the position of a gene along a replichore between the origin and terminus of replication can affect its level of expression simply due to copy number effects: in rapidly-growing bacteria, there are more copies of genes close to the origin than there are copies of genes near the terminus [27–29]. However, gene position may also influence expression due to variations in DNA topology around the chromosome, a proposal that is supported by experimental evidence [30–33]. In addition to location, gene orientation is also thought to avoid head-on collisions between the moving replication fork complex and transcription complexes, especially in the cases of heavily transcribed genes [34]. Furthermore, essential genes are preferentially coded on the leading strand [35]. Inter-gene communication is likely to be an important factor underlying the ability of different gene products to contribute to the same cellular process and/or to regulate each other's expression. Gene networks linked by diffusible gene products (RNA or protein) are probably affected both by intergenic distances along the chromosome and by distances between different segments of the folded chromosome [5–10]. For example, gene product diffusion rates in the crowded cytoplasm influence gene–gene communication [36, 37]. These may account for the presence of statistically co-located (SC) genes whose products contribute to common cellular functions or which cross-regulate one another's expression. Such SC genes occur with a regular periodicity of 117 kbp along the toroidally wound chromosome, placing them immediately above or below one another in the toroid in ways that facilitate gene–gene communication over very short distances [10].

In this study, we performed what is to the best of our knowledge the first investigation of the genome-wide distribution of transcription machinery and transcript output in the context of the macrodomain structure of the *S.* *enterica* serovar Typhimurium chromosome. Our data illustrate shifting patterns of RNA polymerase (RNAP) and sigma factor localization as a function of growth stage, and show that assumptions based on gene dosage in growing cells are an unreliable guide to predicting gene outputs.

## Methods

The 4 878 012 bp chromosome of *S*. *enterica* serovar Typhimurium SL1344 was divided into six even-sized domains of 813 002 bp, oriented such that the Ori domain was centred on the origin of replication (position 4 105 236). Similarly, the 4 639 675 bp *E. coli* K-12 substrain MG1655 chromosome was divided into six even-sized domains of 773 279 bp, oriented such that the Ori domain was centred on the origin of replication (position 3 923 883). Genes that overlapped domain boundaries were assigned to the domain that contained the majority of the gene (Table S1, available with the online Supplementary Material). Transcripts-per-million counts from published data [38, 39] were summed for all protein-encoding genes in each domain. Culture conditions were as in our previous work [39], except that in the present study we used the terms EP (exponential phase), LEP1 (late exponential phase 1) and LEP2 (late exponential phase 2) instead of EEP (early exponential phase), MEP (mid exponential phase) and LEP, respectively, to reflect the physiological changes occurring in growth in Luria-Bertani (LB) medium [40].

Chromatin immunoprecipitation (ChIP) experiments were conducted as previously described [38], using the following monoclonal antibodies from Neoclone: RNAP RpoB (W0001), RpoD (W0004), RpoH (WP006), RpoE (WP007), RpoN (W0005). Transcriptomic and ChIP-microarray (ChIP-chip) analyses were conducted as previously described [41]. RNAP and sigma factor binding sites were determined as those appearing in both of two independent biological replicates [38, 41]; peaks identified in only one replicate experiment represent false positives or weak protein binding sites; thus, our conservative approach of considering only shared peaks reduces the chance of analysing false-positive peaks. For RpoE, RpoH and RpoN datasets, ChIP-chip was also conducted on cells deleted for these sigma factors to identify background peaks that were subtracted from wild-type ChIP-chip datasets. The microarray datasets have been deposited at the National Center for Biotechnology Information Gene Expression Omnibus database, accession numbers GSE97283 (ChIP-chip data) and GSE97161 (transcriptome data); lists of protein–DNA binding sites have been provided as supplemental materials in Tables S2–S6. Unbiased motif searching in the DNA binding sites was conducted as previously described [41].

## Results and Discussion

### Domain structure and distribution of transcription in the *S. enterica* chromosome

The chromosomes of *E. coli* MG1655 and *S. enterica* serovar Typhimurium SL1344 are very similar in size, gene content and gene order (Fig. S1). The gene insertions and deletions that have occurred since the divergence of *E. coli* and *S. enterica* from their last common ancestor are evenly distributed around their chromosomes [18]. There is a large inversion encompassing the replication terminus, but this is wholly contained within the Ter macrodomain (Fig. 1a). The high degree of chromosome structural similarity makes it reasonable to apply to the *S. enterica* chromosome the broad outlines of the macrodomain organization of *E. coli*. To observe genome-wide transcriptional activity in the context of chromosome macrodomain architecture, we divided the *S. enterica* chromosome into six equal-sized zones that were approximately equivalent to the six domains of the *E. coli* chromosome elucidated by Valens *et al*. [24] (Fig. 1a). We then examined the spatial distribution of genes encoding mRNA in multiple growth phases in the model pathogen *S. enterica* serovar Typhimurium. This was done by comparing whole-genome tiled oligonucleotide microarray data to cDNA sequencing (RNA sequencing; RNA-seq) data to quantify transcripts from protein-encoding genes in two phases of growth, late exponential phase (OD_600_ 0.3) and early stationary phase (OD_600_ 2.0) in rich medium (LB; 5 g NaCl l^−1^). Quantification of transcripts by microarray and RNA-seq methods yielded similar measures of gene expression, with remarkably similar transcriptional peaks being observed in late exponential phase (Fig. 1b, c).

Regions of high and low transcriptional output were distributed around the chromosome in both exponential growth and stationary phase. An absence of local uniformity indicated that the putative six macrodomains did not define cohesive regions of transcriptional output. During rapid growth in exponential phase, the highest transcriptional peak was in the NSL domain and corresponded to a cluster of ribosomal protein genes between *rplQ* (SL3381) and *rpsJ* (SL3408) (Fig. 1b, c). The mRNAs from these *rps* and *rpl* genes were significant outliers among the very high number of transcripts originating from all NSL genes.

**Fig. 2.**
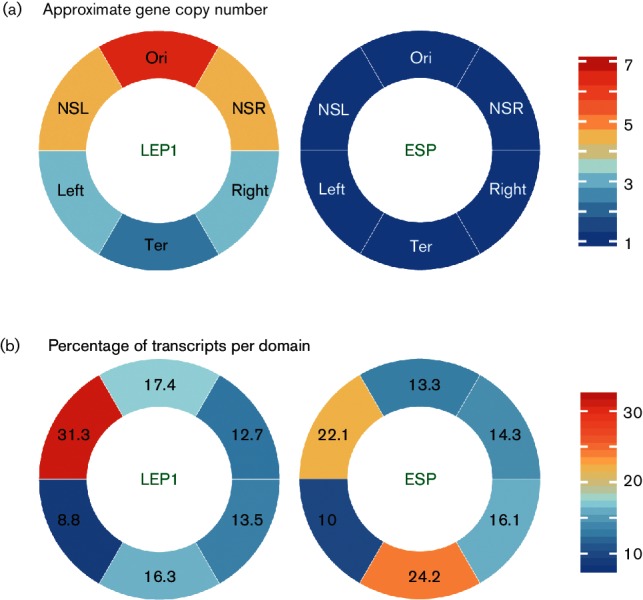
Gene copy number and transcription output per chromosomal domain. (a) The approximate numbers of gene copies in each of the chromosomal domains is shown for an exponentially growing (left) and a stationary phase (right) cell. The red–blue spectrum represents a sliding scale of gene copies ranging from high (red) to unity (blue). Gene copy numbers can be estimated by formulae derived from experiments in *E. coli* B and *E. coli* K-12 [43]. Considering the similar size of the *S*. *enterica* serovar Typhimurium and *E. coli* genomes, the similar generation times of *S*. *enterica* serovar Typhimurium and *E. coli* during growth in rich medium (LB), and that experimentally-determined relationships between growth rate and cellular parameters hold constant across many measurements [42, 43], here we present the mean gene copy numbers in each *E. coli* domain to approximate *S.* *enterica* serovar Typhimurium gene copy numbers in exponential and stationary phases of growth. The higher numbers of gene copies closer to the origin of replication are predicted to elevate transcription from the Ori domain compared to the Ter domain. LEP1, Late exponential phase time point 1; ESP, early stationary phase; see Fig. 3(a) for a growth curve. (b) mRNA abundance is expressed as a percentage of total transcripts per chromosomal domain for *S.* *enterica* serovar Typhimurium. The segments in this panel correspond to the domains named in (a). The red–blue spectrum represents a sliding scale of transcription outputs ranging from high (red) to low (blue).

The hypothetical six-domain organization provided a useful framework within which to identify broad patterns in genome-wide transcriptomic and ChIP data in *S.* *enterica* serovar Typhimurium. It also allowed the mRNA output to be assessed in the context of expectations arising from gene-dosage effects on transcript abundance. When a chromosome is replicating as fast as possible, a fresh round of replication will initiate before the previous copy is complete. Thus, a chromosome can have up to six active replication forks, which creates a 6.5 : 2.2 ratio of Ori-proximal to Ter-proximal gene copies during rapid growth [42, 43] (Fig. 2a). Plotting transcript abundance from protein-encoding genes according to six chromosomal macrodomains showed that neither in rapid growth nor in stationary phase did broad patterns of transcriptional output match the estimated gene-dosage ratios (Fig. 2b). In exponential growth, transcripts from the Ori and Ter macrodomains were very similar in number. In stationary phase, over twice as many transcripts arose from the Ter macrodomain compared to the adjacent Left macrodomain, even though gene dosage across these contiguous domains should be equal at this growth stage. Indeed, the Left macrodomain had the lowest overall transcriptional output at all stages of growth (Fig. 2b), despite containing the *rrsG* ribosomal operon at its boundary with the NSL domain (Fig. 1a). This region of the chromosome is unusual in having a G+C content that is above average for the *S.* *enterica* serovar Typhimurium genome [44] and in *E. coli* having DNA with less negative supercoiling than the rest of the chromosome in both the exponential and stationary phases of growth [33].

### Distribution of transcript abundance across growth phases

**Fig. 3.**
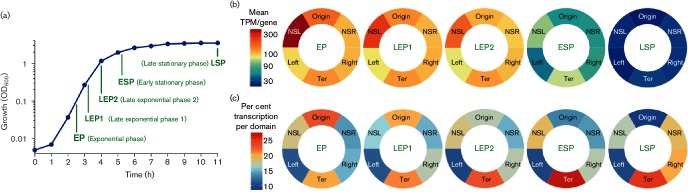
Transcript abundance per chromosomal domain as a function of growth stage. (a) A growth curve for *S.* *enterica* serovar Typhimurium strain SL1344 growing in aerated LB. Vertical lines along the curve indicate the time points at which samples were taken. (b) A summary of transcript abundance for the six chromosomal domains at each of the five sampling points shown in (a). Transcripts per million (TPM) are described in the Fig. 1 legend. (c) The same transcript abundance data in (b) plotted as per cent in each domain, but with the transcripts from ribosomal protein genes *rps–* *rpl* (SL3381–3408) subtracted from the NSL domain. The spectrum at the left of (b) indicates the average TPM per gene in each domain and the spectrum at the left of (c) indicates the percentage of total transcript abundance in each domain ranging from high (red) to low (blue).

Transcript abundance was compared between five time points in batch culture in LB: exponential phase, late exponential phase 1, late exponential phase 2, early stationary phase and late stationary phase (Fig. 3a). RNA-seq datasets were normalized to one million transcripts, enabling direct comparisons between samples and time points, and the quantification of a gene’s expression in terms of genome-wide transcriptional output, including rRNA and small RNA species. As cell division slowed and stopped, transcription shifted from protein-encoding genes to small RNAs, resulting in a genome-wide 10- to 30-fold decrease in mRNA levels (Fig. 3b). To better visualize the relative transcriptional output from each domain through growth, we removed the *rps–rpl* genes (SL3381–3408) in the NSL domain from the dataset because these 28 genes alone account for 22 % (EP), 19 % (LEP1) and 14 % (LEP2) of genome-wide transcription from protein-encoding genes, corresponding to 61 % (EP), 58 % (LEP1) and 48 % (LEP2) of transcripts originating from the NSL domain. Fig. 3(c) shows that even by LEP1 (OD_600_ 0.3), the Ter domain had surpassed the Ori domain for transcriptional output. In stationary phase, more transcripts originated from the Ter macrodomain than from any other domain (Fig. 3c). Overall, patterns of transcript abundance around the chromosome were not in agreement with expectations based on a direct positive correlation between gene dosage and transcript abundance.

### RNA stability may counterbalance gene dosage effects during fast growth

**Fig. 4.**
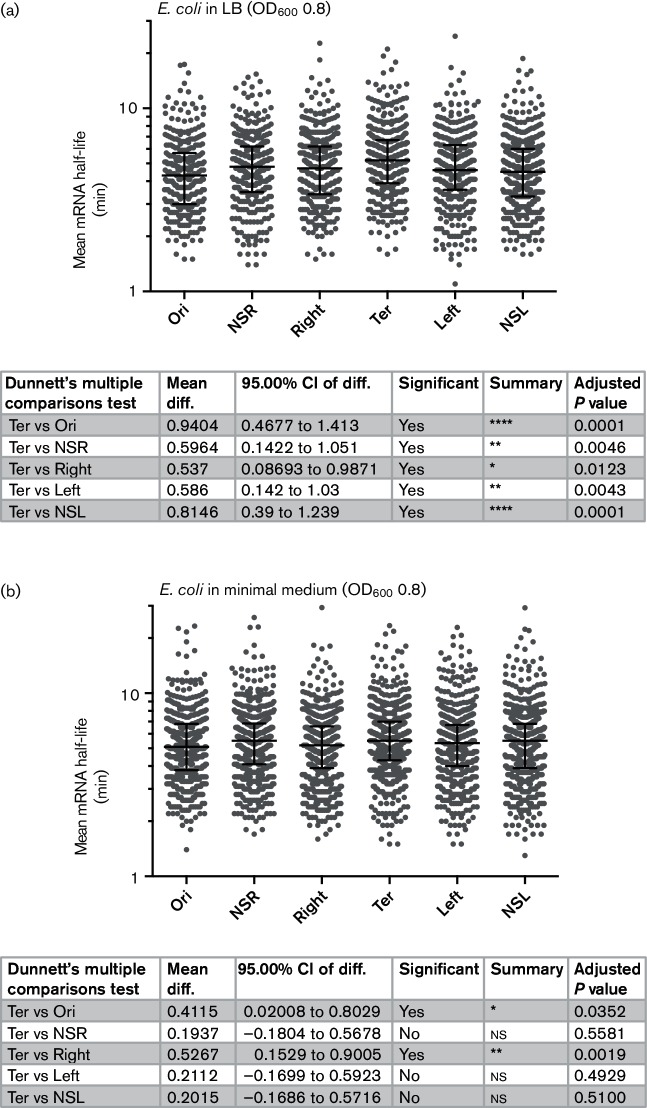
Transcript stability as a function of chromosomal domain. Data from *E. coli* strain MG1655 are presented for cultures grown in LB to OD_600_ 0.8 (a) or in M9 minimal medium with glucose to OD_600_ 0.8 (b). Lines in each scatter plot indicate the median and interquartile ranges. A statistical analysis of the data is presented in the table below each panel together with a statement of statistical significance.

The observation that transcripts from the Ter domain were more abundant than expected from the gene-dosage effect prompted us to consider that transcript stability might not be evenly distributed around the chromosome, and could offset and even counterbalance gene-dosage effects. Analysis of previously published mRNA half-lives in *E. coli* [45] revealed that during growth in LB, RNAs encoded in the Ter domain have a significantly higher mean half-life than RNAs encoded in the other five domains (Fig. 4a). The mean stabilities of transcripts encoded in the other domains were not significantly different from one another.

The biological imperative to conserve energy in nutrient-poor conditions predicts that RNA turnover will be reduced during growth in a minimal medium. Consistent with this, RNAs across the genome were significantly more stable in slower growth in M9 compared to LB (Fig. 4b). Ter domain RNAs were still more stable than Right and Ori domain RNAs, but not significantly different from the other domains in nutrient-limited conditions. Data from *Caulobacter crescentus* suggest that both mRNA production and decay are compartmentalized by the organization of the chromosome [36]. In *S.* *enterica* serovar Typhimurium, the macrodomain structure of the chromosome may provide a framework within which mRNA processes are spatially and temporally organized.

### Distribution of RNAP

**Fig. 5.**
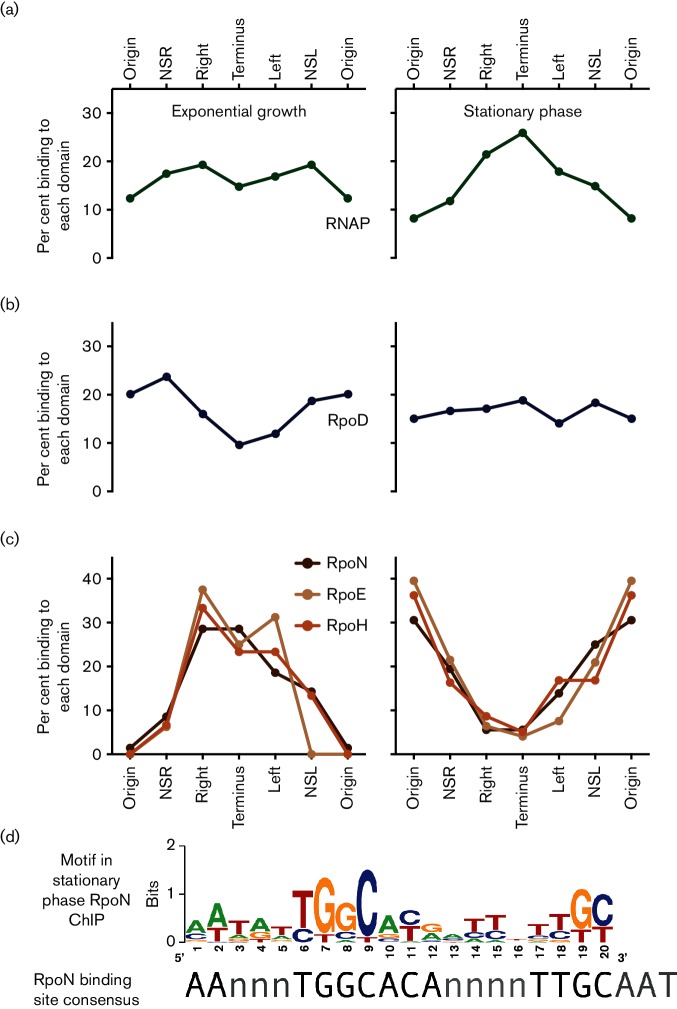
Distribution of the transcription machinery across the six chromosomal domains. (a) The distribution pattern for RNAP in exponential (left) and early stationary phase (right). The per cent total binding in each domain is calculated by considering only ChIP DNA-binding peaks appearing in both of two biological replicates. (b) Binding of the housekeeping sigma factor RpoD across each of the six chromosomal domains in exponentially growing (left) and stationary phase (right) cells. (c) The distribution of the alternative sigma factors RpoE, RpoH and RpoN across the six chromosomal domains in exponentially growing (left) and stationary phase (right) cells. The colour code for each sigma factor is given in the key. (d) Unbiased motif searching of RpoN binding sites identified by ChIP-chip identified sites matching the RpoN binding site motif.

We hypothesized that the transcription machinery (RNAP and its sigma factors) would be concentrated in macrodomains according to transcriptional output, and that transcription machinery would concentrate in different domains as the observed transcriptional output changes as a function of the growth cycle. ChIP-chip analysis revealed that during exponential growth, RNAP was relatively evenly distributed across the chromosome except at the Ori and Ter domains where it was less abundant (Fig. 5a). Conversely, during stationary phase, RNAP was concentrated at Ter and its flanking domains and was further depleted at Ori. The seven highly transcribed *rrs* ribosomal operons and the operon encoding ATP synthase are located chiefly in the Ori and Ori-proximal domains of the *S.* *enterica* serovar Typhimurium chromosome and are known to bind RNAP abundantly during exponential growth [44]. The highly expressed flagella operons are located mainly at the intersection of the Ter–Left domains and they also bind RNAP abundantly during exponential growth [44]. Work with *E. coli* has shown that RNAP is redistributed in stationary phase to non-coding regions of the chromosome [46]. We observed an accumulation of RNAP in the Ter domain in non-growing cells (Fig. 5a). This does not reflect the presence of high concentrations of stationary-phase-specific RpoS-dependent genes within Ter, because the genes of the stationary phase stimulon are evenly distributed around the chromosome [47]. Thus, it is possible that the Ter domain becomes a depot for the storage of DNA-bound RNAP during periods of low transcriptional activity in non-growing bacteria. When *E. coli* grows by doubling every 30 min, 12 % of its 8400 RNAP molecules are free and 28 % are bound non-specifically [48]. Our data suggest that the proportion of non-specifically bound copies increases in stationary phase and that much of this binding occurs in Ter. As the bacterium leaves lag phase at the start of exponential growth, the heavily transcribed operons in the Ori and Ori-proximal domains will create a demand for RNAP. Although the Ter domain is the most distant from Ori on the chromosome circumference, in the folded nucleoid Ori and Ter lie opposite one another [29], perhaps facilitating the transfer of RNAP to those zones where it is needed.

### Distribution of sigma factors

ChIP-chip revealed that during rapid growth and early stationary phase, the primary ‘housekeeping’ sigma factor RpoD (σ^70^) was concentrated in the Ori, NSR and NSL domains (Fig. 5b), and was less abundant at Ter and Left during exponential growth. Overall, this pattern did not parallel the pattern of RNAP distribution at this stage of the growth cycle (Fig. 5a). In stationary phase, RpoD was evenly distributed around the chromosome, due primarily to a depletion at NSR and enhanced occupancy of Ter. Thus, in both stages of growth, the distribution of RpoD did not correspond to RNAP localization.

The alternative sigma factors RpoN, RpoE and RpoH – none of which was active in these culture conditions – displayed a binding pattern that was reciprocal to that of RNAP: they were bound primarily in areas of low RNAP occupancy (Fig. 5c). Thus, there was a clear anti-correlation between the binding pattern of RNAP and those of the alternate sigma factors (RpoE, RpoH and RpoN). Unfortunately, an equivalent anti-RpoS monoclonal antibody was not functional in ChIP-chip experiments. Our results suggest that when they are not participating in transcription initiation, sigma factors nevertheless are associated with transcriptionally non-active regions of the chromosome, in particular, the Left macrodomain (Fig. 5c).

RpoN is structurally distinct from the three RpoD sigma factor family members RpoD, RpoE and RpoH [49]. RpoN binds to its target promoters to create transcription initiation complexes that remain silent until physical contact is made with an appropriately primed enhancer-binding transcription factor [50, 51]. This sigma factor can bind to promoters even in the absence of RNAP core enzyme [52]. Unbiased motif searching of regions bound by RpoN identified a classical RpoN DNA binding site motif in stationary phase binding sites (Fig. 5d), suggesting that this sigma factor was binding specifically though no similar motif was detectable in RpoN binding sites in exponential phase. Thus, even in culture conditions where RpoN is expected to be transcriptionally inactive, RpoN is binding to its target sites in keeping with its known physiological properties.

Previous work with *S.* *enterica* serovar Typhimurium defined approximately 70 binding sites for RpoN. The binding sites were distributed around the chromosome, with more than half being within coding sequences [53, 54]. The Ori macrodomain has the most RpoN sites (17), while the other domains have approximately equal numbers: NSR (8), Right (10), Ter (11), Left (12) and NSL (12). RpoN was not distributed in proportion to the numbers of binding sites per domain (Fig. 5c), suggesting that the Right and Ter domains attracted the protein more abundantly in exponential phase and those sites in the Ori, NSR, and NSL domains were more heavily bound in stationary phase.

In the absence of conditions required for them to activate transcription, RpoE (envelope stress) and RpoH (heat shock) did not bind to DNA showing matches to their binding site consensus sequences, indicating that binding may have been non-specific. Genes belonging to the *S.* *enterica* serovar Typhimurium RpoE regulon [55] were more abundant in the Left (9) and NSL (12) domains than in the others: NSR (6), Right (3), Ter (4), Ori (6). *S.* *enterica* serovar Typhimurium genes matching confirmed members of the *E. coli* RpoH regulon [56] were also unevenly distributed among the chromosomal domains: NSR (11), Right (5), Ter (9), Left (3), NSL (11) and Ori (9). Like RpoN, the distributions of RpoE and RpoH showed a strong anti-correlation with RNAP rather than a positive correlation with their target promoters (Fig. 5c). The genes encoding RpoD, RpoE, RpoH and RpoN are located in NSL, a domain that does not correspond with a peak for sigma factor binding at any stage of the growth cycle. The significance of these colocations is unknown at present, but they may facilitate the cross regulation that has been detected between sigma factor genes [51, 55, 56].

### Conclusions

Transcript abundance does not reflect gene dosage either during rapid growth or in stationary phase. Instead, mRNA output displays a domain-specific pattern in which Ter, the macrodomain with the lowest gene dosage, has a higher output than either the Left or Right macrodomains in rapidly growing cells and exceeds that of Ori in non-growing cells. These unexpected findings may reflect differences in mean half-lives for mRNAs expressed in different domains, with extended half-lives associated with Ter transcripts counterbalancing the gene-dosage effect. During rapid growth, transcription and RNAP are not well correlated, probably reflecting the inability of our detection method to distinguish between specific and non-specific RNAP binding; but in stationary phase, transcription and RNAP localize around Ter. The pattern seen in rapid growth may reflect the longer half-life of mRNA originating in Ter, while the stationary phase pattern may reflect a role for Ter as a store of non-transcribing RNAP during periods when the cell is not growing. Although chemostat cultures and synchronized cell studies will be useful to disentangle the effects of growth rate and culture conditions, in the present study cells were grown in rich medium in batch culture conditions to support comparisons with other broad-scale studies conducted in these same growth conditions in *Salmonella* and *E. coli* over the past decades. We anticipate that similar transcriptional and polymerase dynamics will be observed in *E. coli* and other bacteria because the *E. coli* macrodomain structure has also been successfully extrapolated to another enteric bacterium, *Dickeya dadantii* (*Erwinia chrysanthemi*) [32].

Under growth conditions that do not select for their activities, the alternative sigma factors RpoE, RpoH and RpoN display a distribution pattern that is the reciprocal of that seen for RpoD and RNAP. The alternative sigma factors bind to chromosomal domains that are transcriptionally quiescent. The consistently low transcriptional output of the Left domain is intriguing as this domain has several unusual traits, including a relatively low A+T base content, a lower propensity to form curved DNA than other domains and low levels of binding by the transcription silencing nucleoid-associated protein H-NS [44]. Despite little H-NS binding and high levels of binding by RpoD and RNAP in exponential growth, the Left domain produces the fewest transcripts at all stages of growth. Our data suggest that the chromosome is divided into transcriptionally active and inactive zones at different stages of growth, and that the inactive zones store those parts of the transcription apparatus that are temporarily out of use.

## Data bibliography

1. Cameron ADS, Dillon SC, Kröger C, Beran L, Dorman CJ. Gene Expression Omnibus GSE97283 (2017).
2. Cameron ADS, Dillon SC, Kröger C, Beran L, Dorman CJ. Gene Expression Omnibus GSE97161 (2017).

## Supplementary Data

102 Supplementary File 1

## Supplementary Data

300 Supplementary File 2
