# Supplementary figures and images for: Broad-scale redistribution of mRNA abundance and transcriptional machinery in response to growth rate in Salmonella enterica serovar Typhimurium
Source: Microb Genom. 2017 Aug 4;3(10):e000127. doi: 10.1099/mgen.0.000127 (PMC5695205; doi:10.1099/mgen.0.000127)

***E. coli* K12**

NSR Right Ter Left NSL Ori NSR

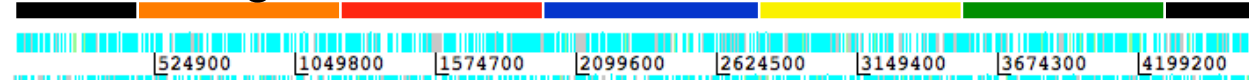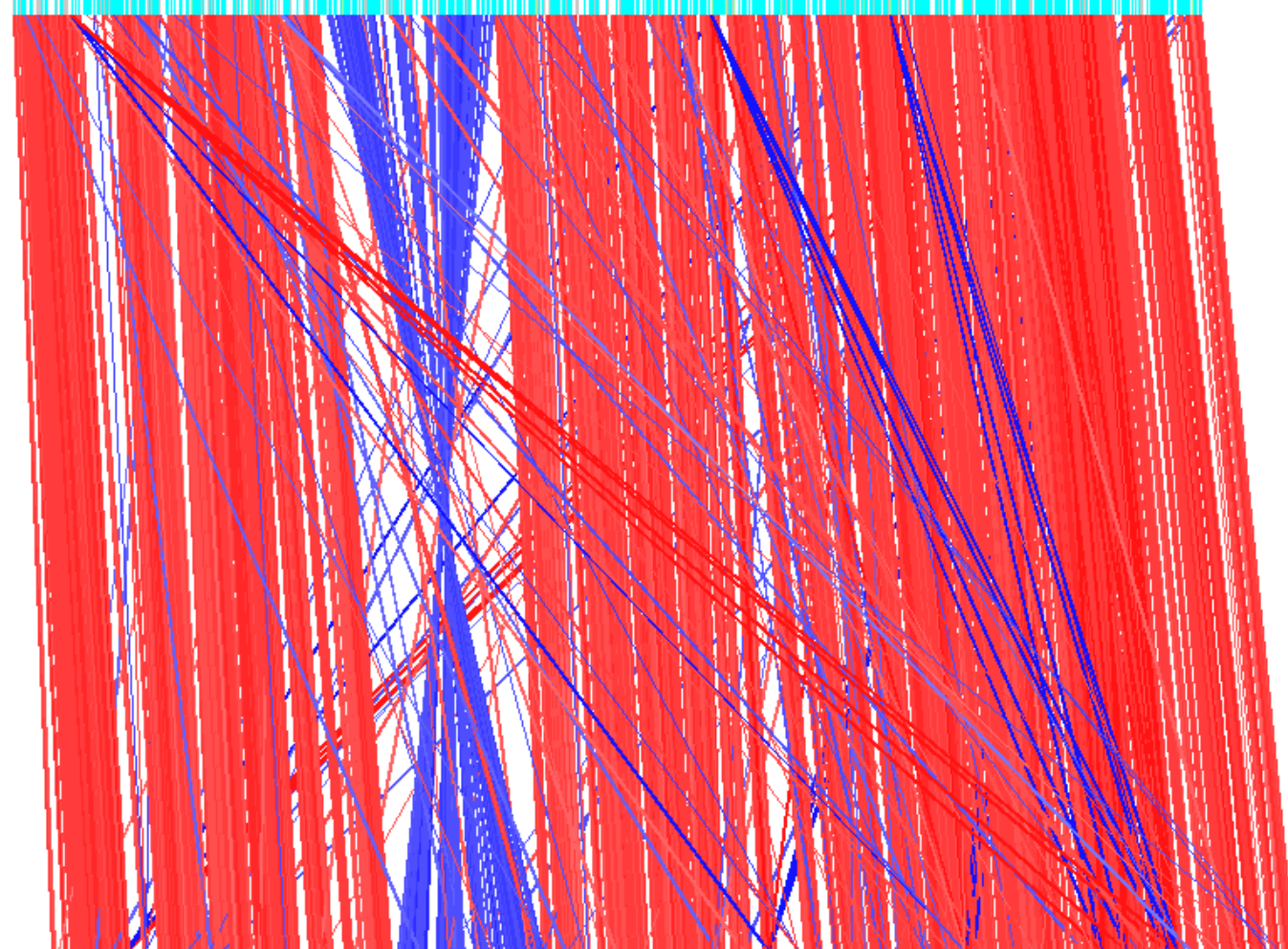

***S. enterica* SL1344**

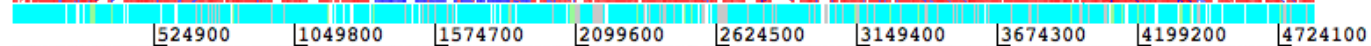

NSR Right Ter Left NSL Ori NSR

Supplement: Supplementary File 2 [file mgen-3-127-s002.pdf]
